# Partners, coordinators and high-level leaders’ perspectives on a consumer and community involvement program in Australia: a qualitative evaluation using template analysis

**DOI:** 10.1186/s12913-025-13685-7

**Published:** 2025-11-25

**Authors:** James Smith, Howard Lance, Susan Hayes

**Affiliations:** 1 School of Population and Global Health, University of Western Australia, Perth, Western Australia Australia; 2 Centre for Precision Health | Collaborative Genomics and Translation Group, Edith Cowan University, Perth, Western Australia Australia; 3 Consumer and Community Involvement Representative, Perth, Western Australia Australia

**Keywords:** Consumer and community involvement, Evaluation, Partners’ perspectives, Qualitative evaluation

## Abstract

A Consumer and Community Involvement program located in Australia supports consumers, community members and researchers to work in partnership with health research priorities, policy and practice. The program is positioned to enhance Consumer and Community Involvement (CCI) at individual, organisational, and system levels. Partners are key to the program’s development, growth and sustainment. This study explored various partner perspectives on their involvement within the CCI program, including individuals (consumers, researchers) organisations (universities, medical research institutes) and the system level (funding organisations) alongside CCI program coordinators (delivering the program) and high-level leaders (decision-makers/executive leaders/directors). Data were collected via two focus groups (10 consumer and community involvement members), 12 interviews (researchers, universities, funding organisations, and medical research institutes), nine qualitative proformas - open-text box surveys (CCI program coordinators (current and previous) and high-level leaders) between March-June 2024. Data were de-identified and Template Analysis was conducted and informed by the Consolidated Framework for Implementation Research. Two themes were produced: (1) ‘Consumer and Community Involvement program structures’ describes the structures of the inner setting (e.g., expertise and support offered, tailored partnerships), and the outer setting (e.g., funding policies, outer consumer and community networks, hub and spoke approach). (2) ‘Processes for connecting consumer/community and researchers’ describe the process (e.g., the communication channels and a bridge between consumer/community members and researchers). This study improves understanding of the planned and tailored structures and processes that embed CCI across individuals, organisations and system levels while highlighting opportunities to strengthen internal consumer connections and diversify consumer representation.

**Supplementary Information:**

The online version contains supplementary material available at 10.1186/s12913-025-13685-7.

## Background

The involvement of consumers and the community in health and medical research is an increasing priority in Australia [1]. Many different terms are used internationally to describe patients and members of the public involved in research, such as ‘patient and public involvement,’ ‘lay representative,’ ‘patient partner’ and ‘public adviser’. The term Consumer and Community Involvement (CCI) is widely used in Australia to describe the involvement of consumers as partners in health and medical research practices, policies, and processes [1–3]. Similarly, various terms and definitions are also noted for consumer and community. Henceforth, consumers are defined as individuals who have lived experience of a health issue. For example, patients, their friends, families, carers and members of the general public. Consumers can also be people who represent the views and interests of a consumer organisation, a community or a wider constituency [4, 5]. The following terms will be used interchangeably, ‘consumers’ and ‘community’.

The overarching aim of health and medical research is to find innovative, breakthrough discoveries for the benefit of all [6]. However, research waste across medical research (clinical or other types) is estimated to consume 85% of the billions spent yearly [7]. CCI can support the aim of health and medical research and help address research waste, by making research more patient-centred, community-relevant and meaningful [8, 9].

CCI is crucial for securing funding, disseminating findings, and increasing research impact [10]. With growing awareness of the need for meaningful CCI, some national and international health research funding bodies are mandating the involvement of CCI across research [2, 11, 12]. Despite widespread agreement that CCI is needed and increased requirements for CCI in health and medical research, as seen in some countries, such as Australia [13], Canada [14], the United Kingdom [15] and the United States [16], the actual approaches to consumer involvement in guidelines vary.

In the Australian context, national accreditation requirements in safety and quality in health care include, as a standard the partnering with consumers, by engaging consumers as partners in planning, design, delivery, measurement and evaluation of systems and services [17]. The National Health and Medical Research Council (NHMRC) in Australia [13] also recognises and supports CCI in research and has issued normative guidance for involving consumer and community members in research. Yet, it is noted that simply providing guidance and information is unlikely to result in any real change [2]. For instance, there is a particular need to detail and understand the many nuances and highly contextual nature of organisations, researchers, consumers, and community members [2]. CCI has been commonly linked to financial barriers, time, funding and staffing issues, and the need to widen consumer representation to include often marginalised voices [8, 18, 19]. In addition, a systematic review on barriers and enablers in CCI echo these findings, further highlighting significant gaps in knowledge about CCI in practice [20] and emphasised the need for additional exploration in how to effectively establish genuine partnerships that involve diversity and inclusion within CCI practice [20].

To create real change, it is therefore important to consider focusing on how to initiate and implement CCI, but this requires an understanding of behaviour and system change [21–23]. For example, researchers, medical research institutes, universities, and funding organisations may all be interested and deeply committed to CCI, but they may, for example, lack clarity on how to engage and connect with consumers, or how to match consumers to researchers, or how to recruit, budget and effectively implement CCI. Consequently, there is overwhelming evidence indicating that the structures and processes involved in initiating and connecting in CCI across the complete spectrum of individual, organisational and system levels are seldom reported and often not well understood [22, 24].

Partnership programs are initiatives that establish and nurture collaborative relationships with various types of partners (e.g., consumer/community, researchers, organisations, funders) [22, 25]. The benefits of partnership programs that incorporate various levels of partnership have been noted [26]. It has been stated that partnership programs should include clear partner agreements tailored to meet the specific needs of each partner [26]. This approach helps create supportive environments that promote the development, growth, and sustainability of a program [26]. Partnership programs, therefore, act as crucial enablers for CCI practice [17]. In CCI, it is also essential that behaviour change occurs at multiple levels to effectively embed and support CCI, which requires commitment at the organisational level, system level, as well as individual researcher and consumer/community level, to ensure effective and authentic involvement in CCI practice [22, 25].

### Program description

The partnership program we have evaluated, the CCI program, directly facilitates the partnership between university organisations, medical and research institutions, grant funding organisations, researchers, and consumers [22, 25]. These different categories of partnership are sought to create mutually beneficial outcomes to improve health and medical research. The partnership program was therefore specifically designed to foster collaboration, build capacity and enhance CCI by matching and connecting consumer/community to researchers at universities (university organisation partners), medical research institutes (medical research institute organisation partners) and funding bodies (funding organisation partners). Henceforth, partners in this particular CCI program are defined and sought at the individual level (consumer/community, researchers), organisational level (universities, medical research institutes), and system level (funding organisations). CCI program’ and ‘program’ will be used interchangeably to refer to this specific CCI program.

The CCI program is grounded on the principle that consumers and community members are active partners with voice, agency, voting power, rights and duties, advocating for what the World Health Organisation calls a “nothing about us without us” approach [27, p. 26]. CCI at its core, offers a unique perspective on all areas of health and medical research. Structures and processes established by the CCI program to involve consumers in health and medical research influence the initial idea and planning, funding and grant applications, the conduct and implementation, and the translation of research findings [28].

The CCI program provides a variety of activities, including online resources and events as well as online and in-person training and workshops. It provides program partners with tailored guidance and support, and a matching service connecting consumers and researchers with relevant involvement opportunities and various CCI methods (Supplementary Material 1). These involvement opportunities are based on the consumer’s skills, lived experiences, interests and goals, and are collected through discussions with the consumer and their referee. The specific criteria for an involvement opportunity are identified by the researcher and CCI program coordinator (advertised by the CCI program on behalf of researchers).

The CCI program provides communication and marketing strategies (including e-newsletters, videos, and social media). The program also delivers free online training and other capacity-building events. For example, the masterclass series (mandatory training currently offered twice yearly) is provided to researchers and innovators to strengthen skills and knowledge of CCI in health and medical research. The CCI masterclass series helps support the translation of CCI methods into best practice processes to promote researchers and consumers engaging with one another.

Considering the various partners involved with the CCI program who are key to the development, growth and sustainability of the program, it is essential to capture their perceptions. This study explored partners’ perspectives of their involvement in the CCI program (which includes consumers, researchers, and partner representatives from medical research institutes, universities and funding organisations), alongside the perspectives of CCI program coordinators (those delivering the program) and high-level leaders (key decision-makers/executive leaders and directors).

## Method

### Study design

Our focus was on practical insights and adopted a pragmatic paradigm [29], which supports drawing on multiple methods to prioritise what actionable strategies work in CCI program practice. Therefore, a qualitative design utilising focus groups, interviews and qualitative proformas was used to explore partner’s perspectives of the CCI program. A defining feature of this study was that consumer focus groups were designed and led by a consumer (HL), with the lead researcher providing essential support (JS). This study adheres to the consolidated criteria for reporting qualitative research (COREQ) reporting guidelines [30].

This evaluation is funded through the Medical Research Future Fund grant number MRF2019278. The study received ethics approval from Monash Health Research Ethics Committee (RES-23-0000-275Q).

### Expert involvement

Various stakeholders with a range of expertise have been involved throughout the research process. Steering committee meetings with consumer and stakeholder representatives were held approximately every three months to inform and guide the evaluation process and review project updates. Regular meetings were held with the project management team (overseeing the project progress) who reviewed and pilot-tested the individual and group interview questions, provided input into the methods of data collection and analysis type and process, and informed the initial template for analysis. Two consumer-investigators (HL & SH) were involved throughout and worked in partnership with JS. Table 1 outlines their consumer involvement throughout the evaluation and represents the agreed level of involvement and specifies the type of involvement of consumers. The consumer-investigators were provided honorariums for their time and expertise.

**Table 1:** Levels of consumer-investigator involvement in the evaluation

Consumer-investigator (involvement at various levels)	Agreed criteria
Embedded within evaluation	Two consumer-investigators (HL & SH) worked alongside JS, evaluating the CCI program. HL and SH were embedded into the research evaluation team to improve the evaluation with their lived experience.
Selection of consumers invited to participate in the focus groups	HL and SH assisted with identifying some of the consumers to be invited to participate in consumer focus group sessions and provided guidance on recruitment.
Undergoing training to lead focus groups	HL underwent training with JS delivering focus group facilitation alongside guides and online video resources on how to conduct focus group sessions.
Leading focus groups	HL was the lead moderator in the discussions and conversations for all focus group sessions supported by JS. HL was well-versed in this role and could frame communication styles from a consumer involvement perspective also utilising his background in education. HL viewed this role as part of building on his existing skill set. JS led all the individual interviews with other Partners.
Reviewing the interpretation and analysis of results	HL and SH have supported JS in reviewing the interpretation and analysis of results, as well as being involved in reviews of the manuscript.
Review and authorship of reports and manuscripts (planned for publication). Other planned co-authorship of academic publications and the evaluation report once the project is finished	Both HL and SH contributions have been recognised and acknowledged and will be co-authors of all related publications.

### Study population

Purposive and snowballing sampling were used to recruit participants. Participants were consumers, academic researchers and members of medical research institutes, universities and funding organisations across the CCI program. In addition, understanding the perspectives and opinions of those at the coalface of the CCI program such as CCI program coordinators (currently employed program coordinators and previously employed program coordinators of the CCI program) who deliver/ed the program and key high-level leaders, provided a wider understanding and triangulation of the partner perspective.

### Data collection

Participants for the interviews and focus groups were emailed an invitation and an explanatory statement for the evaluation to respond to JS about their interest in participating. If there was no response, a second invitation was sent two weeks later. For individual interviews, a mutually agreed time and date were organised. For focus groups, a poll was used to find a suitable meeting time for all to attend. Both focus groups and interviews were conducted online to support accessible involvement. The same study invitation process was followed for the qualitative proformas (open-text box survey) (Supplementary Material 2). Qualitative proformas were managed and distributed via Qualtrics.

Various methods were considered (e.g., interviews, focus groups, and proformas), and different approaches were used across the groups. The different methods were beneficial to ensuring ease of data collection, confidentiality and safety, especially in situations where individual views may have diverged from organisational or group norms. Focus groups were conducted with consumers, led by a consumer-investigator (HL). Interviews with academic researchers, medical research institutes, university organisations, and funding organisations were beneficial to fit in with the various schedules and locations. Proformas were considered the most appropriate method for CCI coordinators and high-level decision-makers, where views, experiences and opinions may have differed. A researcher in a neutral position (JS) oversaw all approaches. JS was the co-facilitator in the focus groups, conducted the interviews, and oversaw the proformas. Informed consent was obtained prior to the online individual and group interviews, and at the start of the qualitative proformas. Data capture events (Table 2) were between March and June 2024 and conducted via two focus groups (approx. 1.5 h each), semi-structured interviews (range 20–56 min) and recorded for transcription purposes. Qualitative proformas are an established qualitative method to support rapid qualitative data collection techniques [9, 31]. They include a minimal number of questions, taking between 15 and 20 min to complete, and encourage detailed answers. Additionally, ample space is provided after each question for free written responses.

**Table 2:** Modes of data collection events

Mode	Description
Online focus groups (via Microsoft Teams)	Consumers were selected from the Customer Relationship Management System (CRM – program membership database), with the inclusion criteria to categorise consumers into two separate focus groups – highly experienced consumers (defined as high involvement in health and medical research), or low-experienced consumers (defined as low involvement of health and medical research). Focus groups captured a range of consumers to incorporate those at different levels, types, etc.
Online interviews (via Microsoft Teams)	CCI program partner names and emails were provided by key informants (program manager and current CCI program coordinators) of the program.
Online qualitative proformas (via Qualtrics)	Names and emails of current and previous CCI program coordinators and high-level leaders were provided by key informants (this included the program manager and both current and past CCI program coordinators) of the program. The qualitative proformas were text-dependent, containing a limited number of questions.

### Data analysis

Template Analysis is an approach in Thematic Analysis [32, 33] and was chosen as it explores patterns within the datasets, and provides a clear, systematic, yet highly flexible approach to data analysis that can be modified throughout, for the needs of any study. Template Analysis involves the development of a coding template and structuring, summarising and thematising data with a subsequent refinement of a coding template, to represent the themes identified in the data [32, 34]. Template Analysis also differs from other forms of Thematic Analysis in that it does not insist on a clear distinction between descriptive and interpretative themes [33]. In addition, Template Analysis was combined with the Consolidated Framework for Implementation Research (CFIR) [35], incorporating the updated CFIR (2.0) domains and was therefore based on previous research [36]. The combination of Template Analysis and the CFIR provides a flexible yet structured approach to coding qualitative data [35]. CFIR has also been used in recent evaluation research to inform complexities and identify the main factors that act as enablers or barriers to implementation [9, 31, 37, 38].

Data analysis was undertaken by JS an experienced qualitative researcher with a background in critical health psychology, complex adaptive systems and implementation science. JS transcribed verbatim all interviews and focus groups, and all data sources were de-identified. Data were analysed together and managed through NVivo 14 [39]. All data sources were coded deductively (top-down/a-priori) to the template and inductively (bottom-up, generated), which involved numerous iterations taken by JS (using the initial template). Modifications included an iterative process through amending, merging, deleting or reintroducing themes, considering instances of different levels of the same phenomenon, and adding constructs within the template, aligning with best practices of Template Analysis [32–34, 40]. Data was sifted, charted, and sorted, which addressed key themes of the template. Once the final version of the template was defined in the light of careful consideration of each data source, it then served to assist in the interpretation and communication of the findings (Supplementary Material 3).

There was no attempt to conduct member checking or to capture data saturation, which is infused with assumptions about reality and knowledge production that sit uncomfortably with this Template Analysis, and this also aligns well with other practices in Reflexive Thematic Analysis [41, 42]. Instead, regular communication took place with the research evaluation team (which included consumers) experienced with CCI to discuss the evolving data interpretation, involving the iterative process and the findings. Regular meetings also took place on the research process with a wider research group (including consumers). All descriptive statistics that characterised the study population were analysed with Stata (version 18.0).

### Quality checks and rigour

The quality checks [43] and rigour followed are demonstrated in Table 3.

**Table 3:** Quality checks

Quality checks	Establishing quality
Trustworthiness	All individual and group interviews, and qualitative proformas, allowed for the expression of perspectives and experiences. Specifically, focus groups were conducted and moderated by a consumer-investigator and an assistant moderator (lead of the evaluation) to enhance conversations and the sharing of views and sentiments.
	Triangulation of findings across multiple data sources.
Transferability	The study design, context and participants are outlined in detail.
	Purposive and snowballing strategies were used to select a diverse sample of relevant participants.
Confirmability	Keeping a reflexive Journal (endeavouring to remain open to participants’ accounts and making a determined effort to step outside of the Researcher role).
	Quotes were included to support themes and sub-themes.
	The themes and sub-themes were discussed with the evaluation team members and presented as presentations to the wider team.
Dependability	The Research audit trail was maintained, including field notes (made after data capture events) and reflexive journaling.
	An audit trail was kept on the template analysis keeping successive versions within NVivo 14.

## Results

Table 4 outlines the diverse range of partners who took part in the evaluation and response rates.

**Table 4:** Data capture events and response rates

Data capture event	Response Rate	Number of participants (*N*)	Partner type per data capture event (*n*)			
Focus Groups	63%	10	*n* = 5 Group [1] Highly- experienced consumers	*n* = 5 Group [2] Low-experienced consumers		
Individual Interviews	67%	12	*n* = 4 Academic researchers	*n* = 3 Medical Research Institutions	*n* = 3 University organisations	*n* = 2 Funding organisations
Qualitative Proformas	47%	9	*n* = 3 Current CCI program coordinators	*n* = 3 Previous CCI program coordinators	*n* = 3 High-level leaders	

Note: Response rates - Focus groups: 16 consumer/community members were invited and 10 participated (63% response rate). Individual Interviews: 18 were invited and 12 participated (67% response rate). Online qualitative proforma: 19 were invited and 9 participated (47% response rate). The overall response rate was 59% for the qualitative data capture events combined

There were a total of 31 participants (highly experienced focus group 1, low-experienced focus group 2, individual interviews and qualitative proformas) with the majority of the sample aged between 45 and 60 years old (48%). More women (84%) than men (16%) participated. Most of the participants were born in Australia and had between 1 and 5 years of experience in the CCI program (Table 5).

**Table 5:** Demographic data

Demographic data	Focus group [1] *n* (%)	Focus group [2] *n* (%)	Individual Interviews *n* (%)	Qualitative proforma *n* (%)	Total *N* (%)
**Age (years)**					
26–44	2 (40.00)	1 (20.00)	3 (25.00)	3 (33.33)	9 (29.03)
45–60	1 (20.00)	2 (40.00)	8 (66.67)	4 (44.44)	15 (48.39)
61–75	0 (0.00)	1 (20.00)	1 (8.33)	1 (11.11)	3 (9.68)
76+	2 (40.00)	1 (20.00)	0 (0.00)	0 (0.00)	3 (9.68)
Missing	0 (0.00)	0 (0.00)	0 (0.00)	1 (11.11)	1 (3.23)
Total	5 (100.00)	5 (100.00)	12 (100.00)	9 (100.00)	31 (100.00)
**Gender**					
Male	0 (0.00)	1 (20.00)	3 (25.00)	1 (11.11)	5(16.13)
Female	5 (100.00)	4 (80.00)	9 (75.00)	8 (89.89)	26 (83.87)
Non-binary	0 (0.00)	0 (0.00)	0 (0.00)	0 (0.00)	0 (0.00)
Prefer not to answer	0 (0.00)	0 (0.00)	0 (0.00)	0 (0.00)	0 (0.00)
Total	5 (100.00)	5 (100.00)	12 (100.00)	9 (100.00)	31 (100.00)
**Identifying as Aboriginal and/or Torres Strait Islander origin**					
No	5 (100.00)	5 (100.00)	0 (0.00)	9 (100.00)	19 (61.29)
Yes, Aboriginal	0 (0.00)	0 (0.00)	0 (0.00)	0 (0.00)	0 (0.00)
Yes, Torres Strait Islander	0 (0.00)	0 (0.00)	0 (0.00)	0 (0.00)	0 (0.00)
Yes, Aboriginal and Torres Strait Islander	0 (0.00)	0 (0.00)	0 (0.00)	0 (0.00)	0 (0.00)
Missing	0 (0.00)	0 (0.00)	12 (100.00)	0 (0.00)	12 (38.71)
Total	5 (100.00)	5 (100.00)	12 (100.00)	9 (100.00)	31 (100.00)
**Identifying with having a disability**					
No	3 (60)	3(60)	0 (0.00)	7 (77.78)	13 (41.94)
Yes	2(40.00)	1(20.00)	0 (0.00)	0 (0.00)	3 (9.68)
Prefer not to answer	0 (0.00)	1 (20.00)	0 (0.00)	0 (0.00)	3 (9.68)
Missing	0 (0.00)	0 (0.00)	12 (100.00)	0 (0.00)	12 (38.71)
Total	5 (100.00)	5 (100.00)	12 (100.00)	9 (100.00)	31 (100.00)
**Country of birth**					
Australia	3 (60.00)	4 (80.00)	0 (0.00)	7 (77.78)	14 (45.16)
England	1 (20.00)	1 (20.00)	0 (0.00)	0 (0.00)	2 (6.45)
Scotland	1 (20.00)	0 (0.00)	0 (0.00)	0 (0.00)	1 (3.23)
Prefer not to say	0 (0.00)	0 (0.00)	0 (0.00)	1 (11.11)	1 (3.23)
Other	0 (0.00)	0 (0.00)	0 (0.00)	1 (11.11)	1 (3.23)
Missing	0 (0.00)	0 (0.00)	12 (100.00)	0 (0.00)	12 (38.71)
Total	5 (100.00)	5 (100.00)	12 (100.00)	9 (100.00)	31 (100.00)
**Self-reported years of experience in CCI**					
Less than 1 year	0 (0.00)	1 (20.00)	0 (0.00)	2 (22.22)	3 (9.68)
1–5 years	1 (20.00)	2 (40.00)	5 (41.67)	2 (22.22)	10 (32.26)
6–10 years	2 (40.00)	1 (20.00)	4 (33.33)	1 (11.11)	8 (25.81)
More than 10 years	2 (40.00)	1 (20.00)	3 (25.00)	4 (44.44)	10 (32.26)
Total	5 (100.00)	5 (100.00)	12 (100.00)	9 (100.00)	31 (100.00)
**Self-reported years of experience in the CCI program**					
Less than 1 year	0 (0.00)	1 (20.00)	0 (0.00)	2 (22.22)	3 (9.68)
1–5 years	2 (40.00)	3 (60.00)	9 (75.00)	3 (33.33)	17 (54.84)
6–10 years	2 (40.00)	1 (20.00)	3 (25.00)	2 (22.22)	8 (25.81)
More than 10 years	1 (20.00)	0 (0.00)	0 (0.00)	1 (11.11)	2 (6.45)
Missing	0 (0.00)	0 (0.00)	0 (0.00)	1 (11.11)	1 (3.23)
Total	5 (100.00)	5 (100.00)	12 (100.00)	9 (100.00)	31 (100.00)

Two themes and related sub-themes (see Table 6), illuminate partners’ perceptions of working in partnership with the CCI program. ‘CCI program structures’ showed factors related to the inner setting structures (e.g., the internal networks and operational aspects that influence implementation) and outer setting structures (e.g., the level at which the CCI program is networked with external entities).‘Processes for connecting consumer/community and researchers’ highlighted the processes influencing the program related to the communication across the network and acting as a bridge between consumers and researchers. All quotes are verbatim. Similar aspects were discussed across all partners and a selection of quotes that represent the themes have been used.

**Table 6:** Themes and sub-themes

Themes	Sub-themes	
1: CCI program structures	1.1 Inner setting structures	1.1.1 Tailored partnership
		1.1.2 Expertise and support
		1.1.3 Mandatory course pathways
		1.1.4 Limited representation
		1.1.5 Limited resources
	1.2 Outer setting structures	1.2.1 Funding policies
		1.2.2 Outer CCI networks
		1.2.3 Hub and spoke
2: Processes for connecting consumer/community & researchers	2.1 Communication channels	2.1.1 Online modes of communication
	2.2 A bridge between consumer/community and researchers	2.2.1 Smoothed the way
		2.2.2 Independent mediator

## Theme 1: CCI program structures

CCI program structures covers the inner and outer setting structures. The inner setting structures cover internal structures and operational aspects. The outer setting structures outline the outer setting layers and outer partner networks.

### Sub-theme 1.1: inner setting structures

Inner setting structures are operational aspects that influence program implementation. This sub-theme covers tailored partnerships, expertise and support, mandatory course pathways, limited representation, and limited resources.

#### Tailored partnerships

Tailored, bespoke partner agreements were noted to be beneficial in outlining the expectations, roles, and responsibilities of CCI. Most of the partner agreements included CCI program coordinators being embedded within partner sites, which assisted partners with direct access for their CCI queries.

*The right relationship [partnership agreement] was formalised through a contract and that’s when [CCI program coordinator]…started sitting with us*,* with our office a couple of days a week* [Funding Organisation]

The partner agreements were described as flexible and some partner agreements were re-considered to find the optimal relationship to suit partner needs.

The management system used within the CCI program houses and assists the partnership membership base. The system is a direct resource and enables matching the research partner with appropriate consumers. Similarly, honorariums were tailored to the role and level of expertise required as part of the set-up structures of the CCI program, with consumers receiving honorarium payments in line with their role as compensation for their time, and for some, the expenses incurred, which will therefore be different for different projects. This ensured the agreed level of involvement of consumers, accounting for such costs in how long and how much consumers will be paid, with this being tailored to the project and consumers’ role and expertise, which will be different for different projects. Honorariums were viewed positively by consumers as a payment for the recognition of sharing one’s lived experience, yet some challenges with signing up to the system were also noted:

*We are always paid for our expertise [Honorarium Payment] [Low-experienced consumer].*

*Signing up on the portal. It’s a bit clunky and…bureaucratic” [Honorariums & CCI program portal]* [Highly experienced consumer].

#### Expertise and support

Expertise refers to both consumer and CCI program coordinator expertise. For example, expertise and support ranged across CCI program coordinator expertise, consumer mentoring and training courses. All partners agreed that the roles and expertise of the CCI program coordinators helped to build, grow and sustain the CCI program network and were key to the partnerships. CCI program coordinators with the appropriate knowledge and skills in providing CCI support ensured the successful implementation of CCI practice. Expertise and support were also recognised as being part of the role of being either an effective CCI program coordinator or an effective consumer:

*Just as in having the ‘right consumer/community member’ involved in a project it is also imperative we have consumer advocates [CCI program coordinators] who hold similar values*,* attributes and skills. The consumer advocates [CCI program coordinators] have to be flexible*,* adaptable*,* consistent*,* persuasive*,* confident and broad in their thinking to be effective* [Previous CCI program coordinator].

Opportunities for growth of the CCI program were highlighted by consumers. Consumers recommended consideration of consumer-to-consumer mentoring (more experienced consumers mentoring less experienced consumers). Some consumer and research partners noted opportunities for growth in new developments in CCI, which are changing roles to consumer-led initiatives and are now becoming more frequent within the CCI space. Consumers suggested running mentorship programs for new consumers, such as ‘train the trainer’ type programs would help develop skills and accommodate new involvement opportunities, methods and strategies:

*You need to get somebody in [consumer/community member]*,* who is maybe one year in somebody who is still a little bit green and maybe they can co-present with somebody else [experienced consumer] and…give some tips about what was helpful for them to get them to 20 years and not burn out* [Low-experienced consumer].

#### Mandatory course pathways

The mandatory sequential CCI program training course pathway (masterclass series) was positively experienced by most partners.

*I noticed a big difference with researchers and helping them when they send you a document to review. Especially their plain language. If they haven’t done the CCI course on how to…write plain language*,* it’s really obvious* [Highly experienced consumer].

In contrast, some of the research partners expressed dissatisfaction with the staged capacity-building approach, perceiving this staged developmental process as not suitable for a research context (nature and scheduling of training) where planning needs to be more flexible:

*Often*,* we have an idea*,* and then we write a grant in a couple of months…so it’s [CCI program] not flexible in that way* [Researcher].

Additionally, suggestions for a more flexible mandatory CCI program pathway (masterclass series would assist in quickly upskilling new staff (new researchers in their organisation) with no background in CCI. At present, these individuals cannot progress without completing the staged competence pathway (only currently being offered twice yearly) and will therefore not have access to the CCI program. This process was stated to not always best suit a researcher’s timeline:

*Is not user-friendly [refers to the CCI program]. But in order to connect with the resource [CCI program]*,* it will take them months and months and they have to go through so many hoops to do the training that*,* that is just not easy to fit into it. That’s not the way research works*,* and so you can’t plan six months ahead cause a grant can be due in three weeks* [Researcher].

Yet, CCI program coordinators called for more recognition of CCI methods from researchers, highlighting this to be a contentious issue:

*I’d also like to see greater respect for our CCI processes from researchers. We are constantly being pushed to amend our methods to the researchers needs which compromises our methods (particularly with events)* [Current CCI program coordinator].

#### Limited representation

An opportunity to further develop the representation of consumers involved in research in terms of diversity of consumers within the CCI program was observed. Often referred to was the frequency of using the same consumers:

*You often see the same people [consumers] across a few things* [Low-experienced consumer]

However, this was not always seen negatively with funding organisations who appreciated working with the same consumers due to connection and their understanding of CCI:

*We tend to use the same ones [consumers] over and over*,* yeah*,* because they are the ones that are happy to do it*,* but also understand what’s going on* [Funding Organisation].

An additional opportunity to develop an internally connected, and diverse consumer representation within the CCI program was reported. Consumers desired to be connected internally to other consumers, but there was no opportunity outside of meetings or events.

*…there could be a large meeting where all the CCI consumers attend rather than limited to this specifically target community conversation. So*,* one for all and even ask other groups to join…A way to connect internally* [Highly experienced consumer].

Similarly, a well-connected, diverse and representative consumer membership was a specific hope, and future opportunity to continue to develop this program:

*I’m keen to see an improvement in the recruiting of attendees to attract a more culturally and linguistically diverse audience. And I’m keen to see the program build its capacity to undertake tailored adaptions of its approaches to better suit different demographics* [Current CCI program coordinator].

*Recruiting CALD [Cultural and Linguistically Diverse] and Aboriginal participants is particularly important. There must also be support to identify and match community/consumers to research projects and/or research groups and ongoing training for both…Being able to deliver some services in languages other than English may also be a useful addition* [High-Level Leader].

#### Limited resources

Various financial aspects, constraints and limitations to resources were noted. In particular, the limited time often allocated with CCI program coordinators (the full-time equivalent (FTE)). Similarly, the program was described by CCI program coordinators as not sustainable long-term due to underfunding, and limited budgeting by partners, thereby impacting ongoing employment contracts for staff [CCI program coordinators] resulting in staff turnover:

*The program is not designed in a way that supports career progression and staff development given the lack of resources available to staff due to underfunding and limited budgeting. These factors surely lead to constant staff turnover which creates a reactive cyclical response with each staffing change* [Current CCI program coordinator].

The dedicated resource of the CCI program coordinator with a designated full-time equivalent (FTE) allows integration into systems and workflows of partner organisations, thus supporting and advancing the development and uptake of CCI in health and medical research. However, all partners desired more CCI program coordinator hours to allow for more FTE hours around the CCI program coordinator position, but partners are required to pay for such additional hours. This problem was often brought up in conjunction with the high cost of CCI activities (community conversations etc.) by researchers, university organisations and high-level leaders. In particular, researchers and university organisations found this to be a challenge.

Likewise, high-level leaders indicated that the current model was not sustainable because it was resource-intensive and expensive to fund. This is also echoed by funding organisations who described the CCI program coordinator position as ‘busy, human work’. It takes a lot of resources and money to supply this type of service in this format. A partner from a medical research institute viewed this as a potentially on-going issue arising from the current rapid expansion of the CCI program due to the increasing awareness and demand for CCI methods. This demand is therefore adding pressure on the CCI program. One partner even suggested to make delivery more efficient and to lessen human staffing costs [funding organisation]. As the CCI program becomes more popular, it is required to service more partners, which will need to be met with additional CCI program coordinators to be hired by the CCI program.

*But when you think about the resource of the CCI program itself*,* it’s just stretched so thin that it’s almost not functional when people need the help* [Researcher]

CCI program coordinators stated that the contracted hours that result from the limited funding received from partners impacts the potential of what could be achieved in CCI delivery.

*Resources*,* including processes and team development*,* is required for things to constantly improve in any program of work/initiative. Team development and individual professional development could lead to better outcomes for the CCI program*,* and this comes with further funding and external support* [Previous CCI program coordinator].

### Outer setting structures

Outer setting structures include external aspects that work on the outer layers of the network (e.g., the level at which the CCI program is networked with external entities). This sub-theme discusses funding policies, outer external CCI networks, and the development of hub and spoke CCI network groups [individual organisations supported by the CCI program to employ their own CCI coordinator/representative and develop their own internal CCI plan in-house].

#### Funding policies

A funding organisation perceived funding as being used as a type of policy-setting tool, with CCI as a must-have if health and medical research is to be funded. An interesting insight was noted from both a university and funding organisation about the role that the CCI program has in both university and funding organisations. This role was perceived as building capacity in CCI methods so that policies from funding organisations and government agencies could actually be implemented. With the CCI program coordinator as a dedicated resource, the CCI program was seen as directly shaping funding programs and therefore shaping policy.

*…so from our perspective having*,* having a coordinator here allows us to consider and embed CCI into the way in which we run our funding programs* [Funding Organisation].

However, one partner expressed confusion over the funding from the wider health network to the CCI program. The sentiment was that the structure and funding need to be communicated more clearly and further clarity and clearer branding from the wider health network and the CCI program is required:

*If you have more clarity are they [referring to the CCI program] part of the [wider health network) or are they separate?…And the website still doesn’t really give you a clear*,* clear vision as to what the [wider health network] is about…I wasn’t aware that…it was actually a Federal funded node initiative perhaps…that’s what I’ve been informed. But again*,* I mean it seems like it could be probably evolved and rebranded to be more relevant*,* but there’s definitely a…brand awareness issue* [Medical Research Institute].

#### Outer CCI networks

The outer CCI network structures of the CCI program refer to consumer/community groups within the CCI program who are highly connected to CCI networks operating externally to the CCI program. This was discussed by both highly experienced and low-experienced consumers alike. Consumers were therefore shown to have strong connections to multiple external (not part of the CCI program) community groups and networks, as demonstrated in the following excerpts:

*Very busy with those two organisations*,* with particularly around infectious diseases and research*,* and also particularly now with the CCI program with a few different things there* [Low-experienced consumer]

*I’m what you call a freelance consumer*,* I suppose because I’ve somehow joined up several other groups [refers to groups outside of the CCI program]* [Highly experienced consumer]

#### Hub & spoke

The term “hub and spoke” was used to describe the CCI program as the hub (CCI program providing tailored support) and individual organisations as the spokes that employ their own CCI representative and develop their own internal CCI plan in-house. This practice was observed positively representing the CCI program successfully contributing to the growth and spread of CCI in health and medical research. This was observed among some partners such as the medical research institute, funding organisations and high-level leaders:

*I would like to see CCI program do more hub and spoke work - i.e. help organisations to establish their own CCI coordinator [CCI program coordinator] or CCI support team*,* with those organisation-based workers then seeking support from a smaller central core team based at [wider health network]. I think this will lead to a more genuine growth and development of CCI across the State that will ultimately be more sustainable than having all services delivered out of CCI program* [High-level leader]

*I think one of the marks is that a lot of people are doing their own programs*,* so there’s like splinter groups. And I think that*,* that’s a real endorsement for how CCI program has spread* [Low-experienced consumer]

Some partners believed that the CCI program’s success led to the development of individual networks and questioned how this new development would ultimately require changes in how the CCI program operates and delivers its services. Concerns were raised about the potential impact on the CCI program, if more partners start to embed CCI from within, with their own CCI program co-ordinator/representative:

*I think they’re in an interesting situation because the more individual organisations employ their own CCI coordinators [CCI program coordinators]*,* the less you need the [wider health network] in its current format in…that environment…I think that’s probably the biggest risk and opportunity for the…CCI program moving forward* [Funding Organisation].

*Some researchers actually have their own CCI Rep consumer with lived experience. They’ve actually built their own little CCI network. Some of it is a little bit of overlap with CCI program*,* but they’re just done the hard jobs*,* just independently. So*,* they’re kind of all set* [Medical Research Institute].

The CCI program was largely commended by partners for directly developing these different spokes and supporting such practices for organisations to grow independently from the CCI program, but also acknowledged the CCI program for the increased awareness of CCI and building capacity by upskilling and educating on CCI across the network. These partners also noted that the CCI program played a crucial role in helping more individual organisations employ their own CCI program coordinators and develop their internal CCI plans, thus directly supported by the CCI program and perceived as part of the CCI program’s role.

*We’ve got a three-year contract with the CCI program to help upscale all of our researchers and develop our own internal CCI plan* [Researcher].

## Theme 2: processes for connecting consumer/community & researchers

Consumers and other partners discussed the processes executed, including planned communication channels to communicate (across the network) involvement opportunities and upcoming events. This was viewed positively by the partners, as a way of implementing and sustaining the CCI program network through executing planned activities and events whereby CCI program coordinators act as a bridge between consumers and researchers.

### Communication channels

This sub-theme covers the need for different modes of communication (e.g., e-newsletters, emails, webinars etc.) as strategies necessary to alert information about CCI and to keep members of the network engaged and updated on events and opportunities provided by the CCI program.

#### Online modes of communication

A good communication platform enabled the dissemination of information to highlight involvement opportunities and spread CCI involvement opportunities:

*They’ve [CCI program] done the dissemination of information very well*,* so I see opportunities popping up in lots of the organisational e-news that I subscribe to. So*,* we’re being bombarded from all over the place. So*,* I think that that might heighten people’s interests* [Low-experienced consumer].

Yet challenges were noted in reaching all the members:

Communicating and getting messages out to all researchers can be really challenging. People receive messages differently. They hear messages differently and they take them on board differently, so we’re always looking to see how can we get the message out. So, from our perspective, we flood all the communication channels, but still people don’t see it and perhaps they just don’t have time or don’t think it’s important [University Organisation].

### A bridge between consumer/community and researchers

This sub-theme covers bridging the gap between consumers and researchers, by how the CCI program ‘smoothed the way’, observing that the CCI program coordinators can act as an ‘independent mediator’ between consumers and researchers.

#### Smoothed the way

The CCI program was viewed as having ‘smoothed’ the way [Highly experienced consumer] to implement CCI in the health and medical research sector:

*I’ve found that when…I’ve attended meetings or things in person*,* the CCI programs typically are well run*,* well organised*,* and very professional* [Highly experienced consumer].

*The staff of CCI program umm*,* are so welcoming and umm really make you welcome*,* whatever the audience is* [Highly experienced consumer].

#### Independent mediator

The CCI program coordinators were described as ensuring both researcher and consumer needs were met. The CCI program coordinators were often portrayed as an independent mediator between the two:

*I’ve certainly been in rooms where I haven’t felt that safe. And*,* felt very disempowered and unable to share an opinion because I feel like I’ll be attacked. But knowing that someone from the CCI program [CCI program coordinator] is there and that they can speak up when*,* when that’s occurring…and ensure that everyone feels safe in that room* [Medical Research Institute].

*One of the Researchers said*,* what about blah blah blah and instantly the CCI employee [CCI program coordinator] said do not fade the health consumers. We wanna hear what the health consumers…say. Now that comment has stayed with me because…I’m here to talk about my experience…to help…not to be told what to say* [Low-experienced consumer].

Differences were noted around the purpose of such meetings. One partner stated that researchers should also be able to intervene and guide community conversations if they are not getting what they need or if the discussions are going off topic.

*One of the challenges is [community conversations]…that the researchers can’t speak. That’s a problem if the conversation isn’t going the way the researchers wanted it to be going*,* there has to be a mechanism live in the moment for the researchers to say ‘I’m not getting what I need out of this. This isn’t the question I want answered’* [University Organisation]

## Discussion

This evaluation examined diverse partners, including consumers, researchers, medical research institutes, universities, and funding organisations. It also gathered insights and opinions from CCI program coordinators and high-level leaders regarding their involvement with the CCI program. The following findings support assertions that partnerships act as supportive environments and are essential to the CCI program’s development, growth, and sustainability.

The first key finding was the identification of the “CCI program structures” (e.g., the inner setting structure supports the operational aspects for working with partners and the outer setting structures of the CCI program show how the program connects with outside groups). The second key finding illuminates the implementation process “processes for connecting consumer/community and researchers” (e.g., the process of smoothing the way for CCI, and being a bridge, with clear communication channels of involvement opportunities connecting the partner network). The findings show individual, organisational, and system change across partners is occurring because it is being supported by the CCI program’s planned and tailored structures, and processes that are embedding CCI into partner roles, decision-making processes, communication methods, and workflows.

The CCI program’s direct influence on policy and governance around CCI, showcases its strong impact through well-developed structures that integrate CCI into partner infrastructures. The insights from partners revealed the benefits of having a dedicated resource, the CCI program coordinator, embedded within the medical research institutes, universities and funding organisations.

This study provides a real-world example of how individual researchers, organisations and systems can engage and connect in CCI, as well as initiate and implement CCI, and validates CCI literature [2, 22, 23, 44]. Initiating and maintaining tailored partner agreements and honorarium payments highlight the important role honorariums/renumeration/compensation play in sustaining consumers as partners [2, 45].

Although the NHMRC provides normative guidance (National Health and Medical Research Council, 2016) for involving consumer and community members in health and medical research, our findings suggest that this information still needs to be synthesised and translated into tangible actions for researchers, organisations and systems to initiate and implement CCI [2, 21, 23, 24]. The CCI program is shifting the dial by moving CCI knowledge from general recommendations to direct action. Specifically, these findings demonstrate that the CCI program partner access to tailored partnerships (e.g., consumers, a dedicated and embedded CCI program coordinator, events such as the masterclass series, tailored support and other activities, etc.) is key to building capacity in CCI education and training. The CCI program provides structures and processes to help researchers understand consumer engagement and build capacity in the CCI sector, fostering CCI skills at any stage to suit partners’ needs. The CCI program helps researchers apply acquired skills to their research ideation, implementation, translation, and grant applications [28]. This movement of CCI knowledge into practice was recognised and noted by consumers who reported a positive and significant difference in the researchers on the CCI program who had taken the e-courses and masterclass series (staged pathway) compared to those researchers who had not taken this staged pathway. However, a particularly interesting tension emerged between researchers’ expectations and the CCI program coordinators’ views on the mandatory training component of the masterclass series (currently only held twice yearly). The masterclass series scheduling appeared to be a barrier to engagement for some of the researchers. This highlights individual differences, which require that, for some researchers, holding masterclasses more frequently than twice a year could ensure appropriateness, given that currently the scheduling and nature of the masterclass series may be preventing some researchers from involving consumers.

The CCI program facilitates connections between consumers/communities and researchers through relationship-building, problem-solving, and ongoing learning, offering involvement opportunities and capacity-building events. The program’s operational structures are organised in a staged manner via the program’s mandatory courses (e.g., e-courses and masterclass series) and showcase the program’s capacity-building approach. The e-courses and the masterclasses, together with tailored support, are some of the ways that the CCI program converts knowledge of initiating and implementing CCI into practice.

The CCI program provides tailored support to meet the unique needs of partners in the research and health service sectors. By being part of the CCI program there is behaviour change in how partners begin to understand and implement CCI in health and medical research and how and what partners may need at different times/phases of their individual development or needs (the staged learning process). Likewise, this aligns with ‘form’ and ‘function’ concepts within implementation science [18, 46], and the CCI program provides a real-world example of this. For example, the range of CCI support and guidance offered by the CCI program is the same for all partners, but the form in which it is delivered is tailored to the needs of each individual partner, as outlined in their specific partner agreement.

Drawing on the implementation science of tailored partnership programs [26, 31], tailored services of the CCI program, provide a structure that is both planned as well as tailored making it both agile and flexible and most importantly, can pivot and adapt to ensure the right solutions are provided to the right partner at the right time [26]. It is important to emphasise that flexibility helps to develop, strengthen and maintain its partner relationships to enrich and sustain the CCI program partner network, supporting broader partnership literature [26]. This is a key strength of the CCI program, alongside strong local network ties being evident, and a strength to program delivery, as well as how the CCI program was networked to external entities such as funding bodies via the CCI program coordinator role. These are examples of how change is happening at a policy level. The CCI program also supports organisations to establish their own CCI program coordinator or CCI support team, with the hub (CCI program) and spoke (partner) approach reported. This could indicate a potential transition phase for this CCI program in how it operates in the future. For example, some university partners were being supported by the CCI program to establish their own individual CCI plan. It was also shown in the use of the CCI program by some more mature or established partners, who were dipping into the CCI program as and when needed. The findings also highlight the concern about limited funding and resourcing for the program, particularly around the need for additional CCI program coordinator time. Yet, the emergence of other organisations establishing their own CCI programs and CCI program coordinator roles suggests a potential shift in the distribution of responsibility and cost. This raises some important points that require further exploration, such as the future sustainability of the program in supporting the broader uptake, and the potential risks (e.g., fragmentation, inconsistent quality) and benefits (e.g., shared ownership, reduced burden) of this more distributed model.

In addition, other connections between the inner and outer settings were noted. Consumers revealed connections to other CCI networks outside of the CCI program. This finding could be linked to the benefits of established structures such as the bespoke matching service, where vacancies are advertised, consumer applicant/s are interviewed and a referee check is carried out on them, with selection emphasis on the connection of the consumer to their outer networks. For example, a clear distinction between consumers’ ‘inner’ networks at the local level (at the CCI program), and ‘outer’ networks (CCI networks outside of the CCI program) was demonstrated in this evaluation by well-connected consumers able to carry with them their own lived and living experience as well as represent the voice of their community in health and medical research projects. Importantly, having consumers who can represent their broader communities is critical [23]. This helps to inform the voice of the consumer, such that the consumer is not just representing their own experiences and opinions, but instead can confidently carry those voices of their external community networks, representing a collective voice within health and medical research.

Effective processes also relied on a strong communication platform for the dissemination of information to highlight involvement opportunities, spread CCI involvement opportunities across its network, and support partners to connect and work alongside consumers, which was a particular strength of the program. The CCI program coordinator role being embedded and onsite to provide tailored support and dedicated resources was a key factor in sustaining partner relationships. This central role fulfilled by the CCI program coordinator contributes to developing, growing and sustaining the CCI network and provides a positive experience across a diverse range of partners. The CCI program effectively provided expertise and organised activities, acting as a bridge to connect researchers with consumers to help partners understand and integrate CCI into their work.

However, there is a need to continue creating an internally well-connected consumer group within the program and grow the network to more diverse consumer groups, expanding particularly in the diversity and representation of CALD and Aboriginal involvement group members and younger individuals. Addressing these community connection needs, recruitment needs, and training needs (delivering training in languages other than English) is recommended and encouraged to ensure all voices with lived experience can be involved and captured in the research process, shaped by historical, social and cultural realities [47]. The extant literature recommends that those seeking to involve consumer and community have a responsibility to recruit individuals from a broad and diverse array of backgrounds to guarantee that the outcomes of health and medical research are widely applicable to improve health and medical research for the benefit of all [8, 45, 48].

Limited resources and funding act to constrain partnerships between consumers and organisations [49]. The underfunding and limited budgeting provided to the CCI program for the program coordinators, is a particular challenge impacting the CCI program’s potential. Specific to this CCI program, adequate external funding should meet increasing CCI demand and provide CCI program coordinators with career opportunities required to ensure quality and knowledgeable staff with job satisfaction. This also links to the human approach of the program, requiring resources to deliver tailored and bespoke CCI expertise and support within its operational structures.

Additional strategies for more rapid acquisition of CCI information and understanding could also be pursued through events and activities with more availability in terms of options for scheduling of events and training. This would cater for an accelerated shift for those researchers who do not have the luxury of planning far ahead. For example, some individual researchers desire to use the CCI program as they need it, but the scheduling can act as a barrier (as per the mandatory and sequential pathway). However, it is important to note that this was not the case for the majority of CCI program partners.

### Limitations and strengths

Online focus groups are not accessible to everyone; those with low digital literacy or no internet may need additional support to participate. We aimed to approach this evaluation in a pragmatic, transparent, and thorough manner. However, this evaluation reflects a specific moment in time (project-based and time-limited). Therefore, the findings should be considered in light of this fact, with clear boundaries and time dependencies.

Efforts were made to capture a range of diversity; however, both the demographic and qualitative data corroborate and report a significant lack of diversity in the program, particularly in areas such as cultural, linguistic, and gender diversity. This lack of diversity reflects the challenges surrounding diversity and inclusion noted in the extant CCI literature [20], highlighting the urgent need for renewed action on this issue within CCI practice.

In the current study, funding organisations (shaping the systems and support around health and medical research through funding, grants, sponsorship, donations and investments) have been referred to as ‘systems.’ It is acknowledged in the literature that systems can encompass broader aspects [22] and embedding CCI at a funding system level remains challenging [2, 23, 25]. This aspect, therefore, has the potential to be expanded and improved upon within future research.

The study employed multi-methods of data collection through focus groups, interviews and proformas. Although it is noted that further probing cannot occur with proformas [31], unlike focus groups and interviews, confidentiality aspects needed to be considered for particular groups directly linked with the program (e.g., CCI program coordinators and high-level leaders). The various approaches also enabled working with some partners schedules. Further strengths include that the consumer focus groups were consumer-led, and consumer-investigators were involved throughout the study in various levels of involvement (Table 1) promoting an example of genuine meaningful partnership.

### Recommendations

Taking action based on evaluation findings is helpful when results are shared quickly. It is important to include rapid feedback loops [50] to share timely information with CCI program decision-makers. Shifting beyond a solely project-based, time-limited nature toward longitudinal evaluations that incorporate rapid-cycle feedback loops at key time-points is an important call to action that can further enhance meaningful engagement within CCI [51].

Given the observable shift in policy and practice across funding bodies to mandate CCI for all health and medical research funding [52], the CCI program will need to adapt and respond to this important sea change, the CCI program will require real-time evaluation data to both manage intended (an instant increase in new partnerships) as well as unintended consequences (a potential to disrupt the existing structures and processes of the program, further limiting resources available to existing partners within the CCI program).

The study findings highlighted various local organisations are now establishing their own CCI programs. Future research is required to review CCI programs to understand what works and what does not, and similarities and differences in approaches locally, nationally, and internationally, to ensure consistent quality.

It is known that various definitions are used for CCI, which can be confusing, since definitions and terms are used interchangeably. A concept analysis is recommended to further review the clarity of terms and provide a clear definition [53–55].

## Conclusion

Lived experiences, values and priorities of consumers/community members are paramount in research, policy and practice. The evaluation exploring various partner perspectives demonstrated that the CCI program is an excellent example of taking decisive action to involve CCI in health and medical research through the lens of individual (consumers, researchers), organisational (university, medical research institute) and system levels (funding policies). Individual, organisational, and system change across partners is happening because of the CCI program’s planned and tailored structures, and processes that positively shape how partners are implementing CCI as a result. The CCI program has established these essential structures and processes for a well-run, well-organised, and highly professional delivery, translating CCI methods by sharing knowledge across its partner network, developing training content to educate partners, and connecting researchers and organisations to consumers. The evaluation revealed a collaborative network with a need to address CCI program partners’ desire for more time with CCI program coordinators, which related to the need for additional external funding in the course of their work to discuss and seek CCI program coordinator advice. The CCI program demonstrated its impact through the masterclass series, disseminating information, and providing expert CCI advice. However, there is an opportunity to grow the network to include more diverse consumer groups. This situation underscores the pressing necessity for a concerted effort to implement meaningful changes related to diversity and inclusion in CCI practice.

Lastly, the future looks promising in terms of the continued development, growth and sustainment of the program, through continuing to support behaviour change at the individual, organisational and system levels to effectively integrate CCI into the conception, delivery, and implementation of health and medical research. A continued keen focus on how and how well implementation of CCI is being achieved in practice will ensure that research dollars are directed towards addressing the most important and pressing needs of consumer/community members, ultimately leading to innovative and ground-breaking discoveries for the benefit of all.

## Supplementary Information

Below is the link to the electronic supplementary material.

Supplementary Material 1

Supplementary Material 2

Supplementary Material 3

## Data Availability

The datasets generated and analysed during the current study are available from the corresponding author on reasonable request. The data are not publicly available due to containing information that could compromise research participant privacy/consent.

## Abbreviations

CFIR: Consolidated Framework for Implementation Research
CCI: Consumer and Community Involvement
NHMRC: National Health and Medical Research Council
