# Supplementary material for: Partners, coordinators and high-level leaders’ perspectives on a consumer and community involvement program in Australia: a qualitative evaluation using template analysis
Source: BMC Health Serv Res. 2025 Nov 25;25:1524. doi: 10.1186/s12913-025-13685-7 (PMC12649027; doi:10.1186/s12913-025-13685-7)
Supplement: Supplementary file 1 — Supplementary Material 1 [file 12913_2025_13685_MOESM1_ESM.docx]

Supplementary Material 1: Examples of Consumer and Community Involvement Methods

| **Examples of CCI Methods** | **Practice Examples** |
| --- | --- |
| Community Conversations | Accessing input from groups of up to 30 people with a specific lived experience to inform researchers about the community’s views on the related topics. |
| Research Priority Mapping Workshops | Explore consumers’ and/or community views as lived experience priorities for research on a particular topic. |
| Document and/or Grant Reviewers | Review documents and/or grants for plain language, readability and inclusion of the lived experience perspective in research ideation, design, implementation and translation |
| Consumer-Investigators or Research Buddies | Using the matching service, consumer-investigators help to provide the link between researchers and those with lived experience as ongoing members of the research team. |
| Consumer/Community Representatives | Teams, groups, panels, and committees to advise and inform research ideation, design, implementation and translation. |
