# Supplementary material for: Partners, coordinators and high-level leaders’ perspectives on a consumer and community involvement program in Australia: a qualitative evaluation using template analysis
Source: BMC Health Serv Res. 2025 Nov 25;25:1524. doi: 10.1186/s12913-025-13685-7 (PMC12649027; doi:10.1186/s12913-025-13685-7)
Supplement: Supplementary file 2 — Supplementary Material 2 [file 12913_2025_13685_MOESM2_ESM.docx]

Supplementary Material 2: Interview guides

Online Focus Group Questions (Consumers only)

1. Let's start by introducing ourselves. Could you inform the group of your name and how long you have been a member of the program?
2. Think back to when you first became part of the program - how did you become aware of the program?

*Prompt:*

- *Why did you first start working with the program?*
- *Why did you get involved with the program?*
- *What attracted or encouraged you to become a member?*

1. How do you connect with other consumers and/or community members and researchers *within and beyond the program?*

*Prompt:*

*How do you find out about projects to be involved in?*

*Such as consumer/community groups you’re involved in within or beyond your program membership*

1. What are the key activities or processes delivered by the program that keep you engaged in the program and what if anything, could be improved upon?

*Prompt:*

- *CCI program coordinators matching services*
- *Community conversation*

*CCI program coordinators to match you and your project with researchers in terms of:*

- *Research Buddy*
- *Document/Grant reviewer*
- *Research Priority Mapping*
- *Training*
- *Regular eNewsletters and promotion of Involvement Opportunities, events, and updates from research sector*

1. What are the strengths of the program that keep you engaged with the program and what are its challenges that lead to non-participation?

*Prompt:*

- *Lived up to your expectations/met your needs throughout your involvement?*
- *What aspects are done well by the program?*
- *For example, highlights of working with the program – details of process, who was involved etc*
- *For example, workshops, training, community conversations?*
- *Are there any challenges regarding working with the program, and/or its membership?*
- *For example, communication with researchers*

1. What has your experience been in the program working with researchers within healthcare improvement settings?

*Prompt:*

- *Increasing confidence in working with researchers?*
- *For example, has your involvement shaped your knowledge/thinking?*
- *Is your involvement in research valued by researchers? If so, how?*
- *Feeling valued, having a voice?*

1. How do you see the program being delivered in the next 5 years?

*Prompt:*

- *What would that look like?*
- *What else could be done?*
- *Who would it involve?*

1. In the time that you have been involved with the program has the consumer engagement actually improved?
2. Finally, use the icon on your screen or just put your thumbs up, if you would recommend the program to other people you know.
3. Is there anything else we haven’t discussed yet that you think is important to share/for us to know?

Interview Questions (Funders only)

1. Could you tell me your name and role and how long you have been a part of the program?

*Prompt:*

- *How does this role interact or contribute to the program/fit the research sector/centre/consumer*

1. Think back to when you first became part of the program. How did your organisation first become aware of the program?

*Prompt:*

- *Why did your organisation first start working with the program?*
- *What attracted or encouraged you/organisation to become a part of the program?*

1. How has the program been embedded in your organisation?

*Prompt:*

- *What opportunities or challenges has this presented?*
- *What structures and processes were put in place to support the program and its expansion?*
- *How did you develop opportunities or navigate challenges to grow the network?*

1. How has your organisation incorporated CCI into your funding/grant schemes and their governance and promotion?

*Prompt:*

- *Information about CCI when scheme was launched?*
- *Information sessions for potential applicants?*
- *[CCI criteria application form]*
- *CCI scoring of funds/grants?*
- *Do they have specific training regarding CCI scoring/criteria for their grant review/assessment panels?*
- *Reporting - progress and final report are you getting people to report it and what has the impact been.*
- *Case studies on CCI in completed grant schemes?*

1. What role/support, if any, has the program played in shaping your funding programs/governance and/policy within your organisation?

*Prompt:*

- *And/or developing CCI policy/governance/promotion and/or information?*
- *Support within and beyond your organisation*
- *Benefits to individual researchers and how has the program benefited you e.g., saved you time sourcing resources*
- *What else is needed?*
- *What are some of the gaps?*

1. What has your experience been working with the program?

*Prompt:*

- *What has worked well (administrative load etc.)? What could be improved?*
- *Researchers/consumers in the program*
- *Benefits/disadvantages*
- *Increasing confidence in working with consumers?*

1. What would you like to see the program delivering in the next 5 years?

*Prompt:*

- *What would that look like?*
- *What should it include? How should it work?*
- *What is currently in place to keep the program sustainable?*
- *Individuals/champions*
- *Systems/processes*

1. Is there anything else we haven’t discussed yet that you think is important to share/for us to know?

Interview Questions (Researchers, University Organisations & Medical Research Institutes)

1. Think back to when you first became part of the program. How did you first become aware of the program?

*Prompt:*

- *Why did you first start working with the program?*
- *Are you still actively involved?*
- *How and why are you still involved?*
- *What attracted or encouraged you to become part of the program?*

1. How has the program been embedded in your research or, organisation, or institute?

*Prompt:*

- *What opportunities or challenges has this presented?*
- *What structures and processes were put in place to support the program and its expansion?*
- *How did you develop opportunities or navigate challenges to grow the program?*

1. How do you connect with consumers and community members locally/nationally? Is it through the program or some other avenue.

*Prompt:*

- *Why do you connect with the program?*
- *What do you gain from this connection to the program?*
- *Such as consumer/community groups you’re involved in within or beyond your program membership?*
- *What is working well, and what could be improved on?*

1. What are your thoughts on the tailored support delivered in the program?

*Prompt:*

- *program coordinators and matching services?*
- *program coordinators to match you and your project with consumers in terms of:*
  - - *Research Buddy*
    - *Document/Grant reviewer Community Conversations*
    - *Research Priority Mapping*
    - *Think broader than this – what processes (e.g., funding, policies etc) what structures (e.g., roles, regular evaluation etc)*
- *Have you utilised any of these tools/supports?*
- *What was your experience like?*
- *How could this process be improved?*
- *What worked well?*

1. What was your experience working with a program coordinator?

*Prompt:*

- *What did it look like, was it helpful and why?*
- *Could it be improved?*
- *Is there additional support you think the program coordinators could provide?*

1. Are you aware of any other resources in relation to the program?

*Prompt:*

- *Other events and resources such as:*
  - *Masterclass series*
  - *Online course*
  - *Community conversations*
  - *Research priority mapping*

1. How has the program influenced your work?

*Prompt:*

- *Benefits as an individual researcher/organisation*

1. How has the program benefited you e.g., saved you time sourcing resources?
2. What has your experience been working with consumers in the program?

*Prompt:*

- *Increasing confidence in working with consumers?*
- *How did this impact your work?*

1. Are there any challenges regarding working with the program, and/or its membership? program coordinators

*Prompt:*

- *Communication with researchers should be helping more with/providing more services and support?*

1. What would you like to see the program delivering in the next 5 years?

*Prompt:*

- *What would that look like?*
- *What should it include?*
- *How should it work?*
- *What if anything else is needed to keep the program sustainable?*
- *What is currently in place to keep it sustainable?*
  - *Individuals/champions*
  - *Systems/processes*

1. Is there anything else we haven’t discussed yet that you think is important to share/for us to know?

Qualitative Proformas

| Qualitative open text-box proformas open-ended question format  [for program coordinators previous and current] |
| --- |
| 1. What are your thoughts on the type of support that you deliver/ed as a program coordinator/consumer advocate? |
| 1. What works or does not work with the program coordinator/consumer advocate role? (clinical staff only) |
| 1. What would you like to see the program delivering in the next 5 years?   [Prompt: Opportunities/improvements/gaps that should be considered] |
| 1. Is there anything else that you think is important to share/for us to know? |

| Qualitative open text-box proformas  [for High-level leaders] |
| --- |
| 1. How and why was the program first established?   [Prompts: What was your role in its establishment? Where did the idea come from? What were the processes and structures involved?] |
| 1. What delights or challenges existed in initially setting up/being part of this program? |
| 1. What do you see as the successful components needed in developing a CCI network? |
| 1. What would you like to see this program delivering in the next 5 years? |
| 1. Is there anything else that you think is important to share/for us to know? |
