# Supplementary material for: Partners, coordinators and high-level leaders’ perspectives on a consumer and community involvement program in Australia: a qualitative evaluation using template analysis
Source: BMC Health Serv Res. 2025 Nov 25;25:1524. doi: 10.1186/s12913-025-13685-7 (PMC12649027; doi:10.1186/s12913-025-13685-7)

Supplementary Material 3: Final template to visualise and assist interpretation and communication of the findings


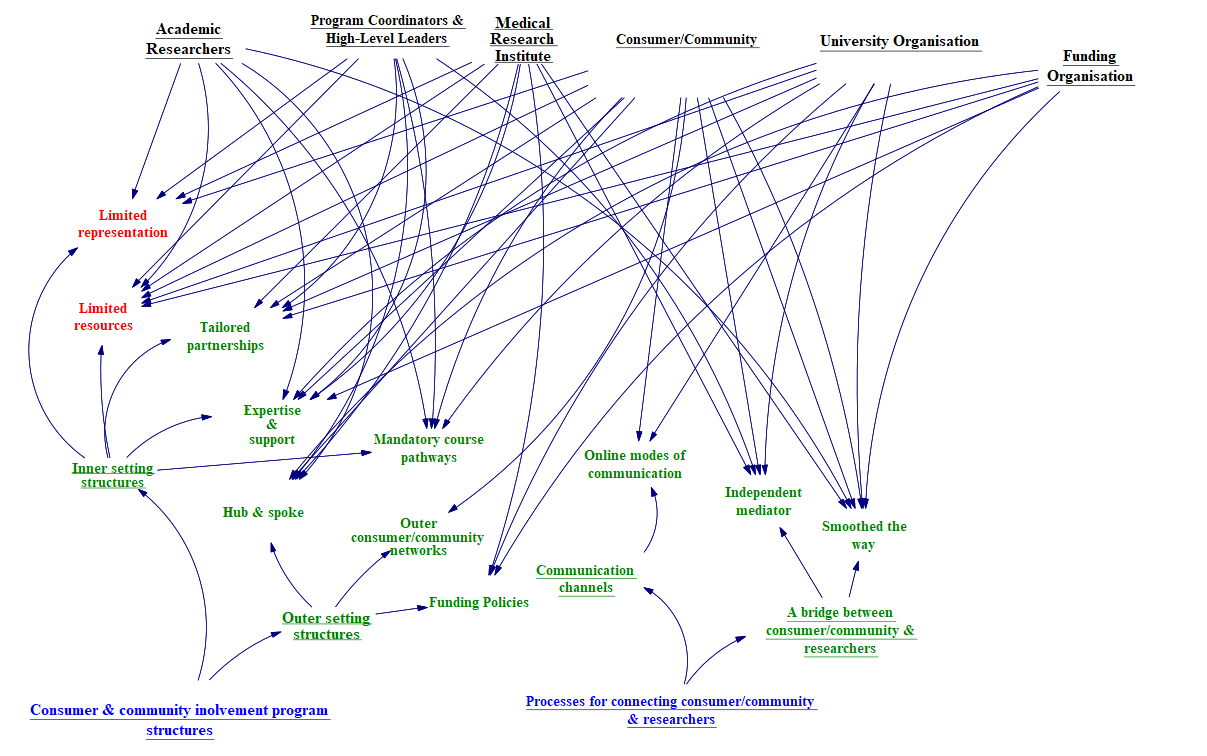

Supplement: Supplementary file 3 — Supplementary Material 3 [file 12913_2025_13685_MOESM3_ESM.docx]
